# Association between histamine-2 receptor antagonists and adverse outcomes in neonates: A systematic review and meta-analysis

**DOI:** 10.1371/journal.pone.0214135

**Published:** 2019-04-04

**Authors:** Victor S. Santos, Marina S. Freire, Ruth N. S. Santana, Paulo R. S. Martins-Filho, Luis E. Cuevas, Ricardo Q. Gurgel

**Affiliations:** 1 Centre for Epidemiology and Public Health. Federal University of Alagoas, Arapiraca, Brazil; 2 Department of Medicine. Federal University of Sergipe, Aracaju, Brazil; 3 Postgraduate Programme in Health Science. Federal University of Sergipe, Aracaju, Brazil; 4 Investigative Pathology Laboratory, Federal University of Sergipe, Brazil; 5 Liverpool School of Tropical Medicine, Liverpool, United Kingdom; Hopital Robert Debre, FRANCE

## Abstract

**Background:**

The use of histamine-2 receptor antagonists (H_2_RA) in neonates is still debated because of possible risk of infection, necrotizing enterocolitis (NEC) and increased mortality.

**Aim:**

To review whether the use of H_2_RA in neonates admitted to neonatal intensive care units (NICU) is associated with infection, NEC or mortality.

**Materials and method:**

We performed a systematic search in PubMed, Web of Science and SCOPUS databases using the terms “histamine-2 receptor antagonists”, “infection”, “necrotizing enterocolitis”, “mortality”, “neonates” and related terms to identify studies published up to April 30, 2017. We included studies conducted in hospitalized neonates and exposed to H_2_RA. The primary outcomes were infection, NEC and mortality. We included reports of infections with clinical signs and positive culture, and NEC according to Bell stages (stage ≥II) based on standardised clinical and radiologic criteria. Among 1,144 studies identified, 10 fulfilled the selection criteria. Information extracted included study design, sample size and number of participants, along with the outcomes of interest. We conducted a meta-analysis of adjusted data and pooled estimates of infection, NEC and mortality are reported as odds ratios (OR) and 95% confidence intervals (95%CI).

**Results:**

Ten studies were analysed. There were substantial associations between H_2_RA and infection (pooled OR: 2.09; 95%CI: 1.35–3.24; P = 0.001) and NEC (pooled OR: 2.81, 95%CI: 1.19–6.64; P = 0.02) but not with the mortality risk (pooled OR: 1.76; 95%CI: 0.50–6.16; P: 0.38).

**Conclusion:**

Current evidence suggests that H_2_RA is associated with an increased risk of infection and NEC, but not with mortality in neonates admitted to NICU. The use of H_2_RA in neonates must be stringently considered when necessary.

## Introduction

Histamine-2 receptor antagonists (H_2_RA) are often prescribed *off-label* to neonates admitted to neonatal intensive care units (NICU) [1] for prophylaxis or therapy of stress ulcers and gastroesophageal reflux disease (GERD). However, the safety and efficacy of H_2_RA in neonates is still debated [2]. This is due to gastric acid secretions being one of the main non-immune defenses against invading pathogens [3] and the sustained inhibition of gastric acid secretions alters the bacterial ecology favoring the gastric colonization of enteric bacteria and may facilitate microbial translocation across the gut barrier because of decreased neutrophil activity [4,5]. Studies have shown an increasing gastric pH within few minutes of H_2_RA administration [6,7], with effects on the H_2_ receptors activation and modelling of the immune responses, especially in the production of inflammatory cytokines [8–10].

A 2014 systematic review of clinical trials conducted in 1 to 15 years old children reported that H_2_RA were effective in reducing GERD signs and symptoms, but did not report adverse effects in a measurable manner, precluding a quantitative analysis on drug safety [11]. Other studies in neonates however have shown that H_2_RA may predispose to infections [3,12–14], necrotising enterocolitis (NEC) [14,15] and death [14,16], but there are no systematic analyses of this evidence.

We conducted a systematic review and meta-analysis to investigate whether the use of H_2_RA in neonates admitted to NICU is associated with an increased risk of infection, NEC and mortality.

## Materials and methods

This study was conducted following the Meta-Analysis of Observational Studies in Epidemiology (MOOSE) statement (S1 File) [17]. Institutional review board approval and informed consent were not required for this systematic review and meta-analysis. A study protocol was designed a priori and was registered in the PROSPERO database (registration number CRD42017060887).

### Search strategy and selection criteria

We performed a systematic review using PubMed, Web of Science and SCOPUS databases to identify studies published up to April 30, 2017 without language restriction. Publications were identified using the terms “histamine-2 receptor antagonists”, “infection”, “necrotizing enterocolitis”, “mortality”, “neonates” and related terms. The full search strategy is described in the S1 Table. Two independent reviewers (MSF and RNSS) screened titles and abstracts for relevance and adequacy and disagreements were resolved by VSS and RQG. The manuscripts selected were read in full to confirm their eligibility and their reference lists were scanned to identify additional studies. We included studies conducted with neonates hospitalized in NICU and exposed to H_2_RA. We excluded studies in infants over 28 days old, those not containing original material or reporting data from ambulatory patients and studies including neonates with infections before initiating H_2_RA, congenital malformations or genetic syndromes, mothers with HIV, rubella, toxoplasmosis, cytomegalovirus or hepatitis B and C.

### Outcomes

The primary outcomes were infection, NEC and mortality. We included reports of nosocomial infections with clinical signs and positive culture. NEC was classified according to Bell stages and included children with Bell stage ≥II [18].

Secondary outcomes included pneumonia, sepsis and urinary tract infections (UTI). For these outcomes, we consider studies that had defined a) pneumonia as the presence of clinical signs associated with positive culture or radiological findings with suggestive signals of pulmonary involvement by infectious agents (persistent infiltrate, consolidation and cavitation) and abnormal laboratory tests; b) sepsis as the presence of signs suggestive of infection associated with a positive blood culture, and c) UTI when there was a positive urine culture together with clinical findings.

We analyzed the mortality at any time during the follow-up period, as reported in the study included in the meta-analysis.

### Data extraction and bias assessment

We used pre-formatted tables for data extraction, including author, publication year, country, study design, sample size, number of participants with infections, NEC or death by H_2_RA exposure. Not all studies reported the absolute numbers of the outcomes and frequencies were calculated from percentages. For articles not available in electronic databases or data unavailable in the articles included, we attempted to contact the authors to obtain relevant information. We had planned to extract data on the exposure time and dosage of H2RA to ascertain duration of exposure and a dose safety gradient; however, it was not possible to obtain the data for meta-analysis. The risk of bias for individual studies was assessed by two independent reviewers using the Newcastle-Ottawa Scale (NOS) [19] and disagreements were resolved by discussion.

### Statistical analysis

We calculated the pooled odds ratio (OR) for the primary and secondary outcomes and used forest plots to present effect sizes with 95% confidence intervals (95%CI). Pooled unadjusted and adjusted estimates were calculated using Mantel-Haenszel and inverse variance methods, respectively. The meta-analysis was performed using random-effects model. Two-tailed P-values <0.05 were used to determine statistical significance. Statistical heterogeneity was assessed using the Cochran Q test [20] and quantified by the *I*^*2*^ index [21]. A subgroup analysis was performed according to the study design (cohort or case-control).

Leave-one-out sensitivity analysis was conducted by omitting one study at a time and examining the influence of each study on the pooled effect size [22]. Analyses were performed using Review Manager 5.3 (Cochrane IMS, Copenhagen, Denmark) and R-3.3.2 software (R Foundation for Statistical Computing, Vienna, Austria).

## Results

**Fig 1:**
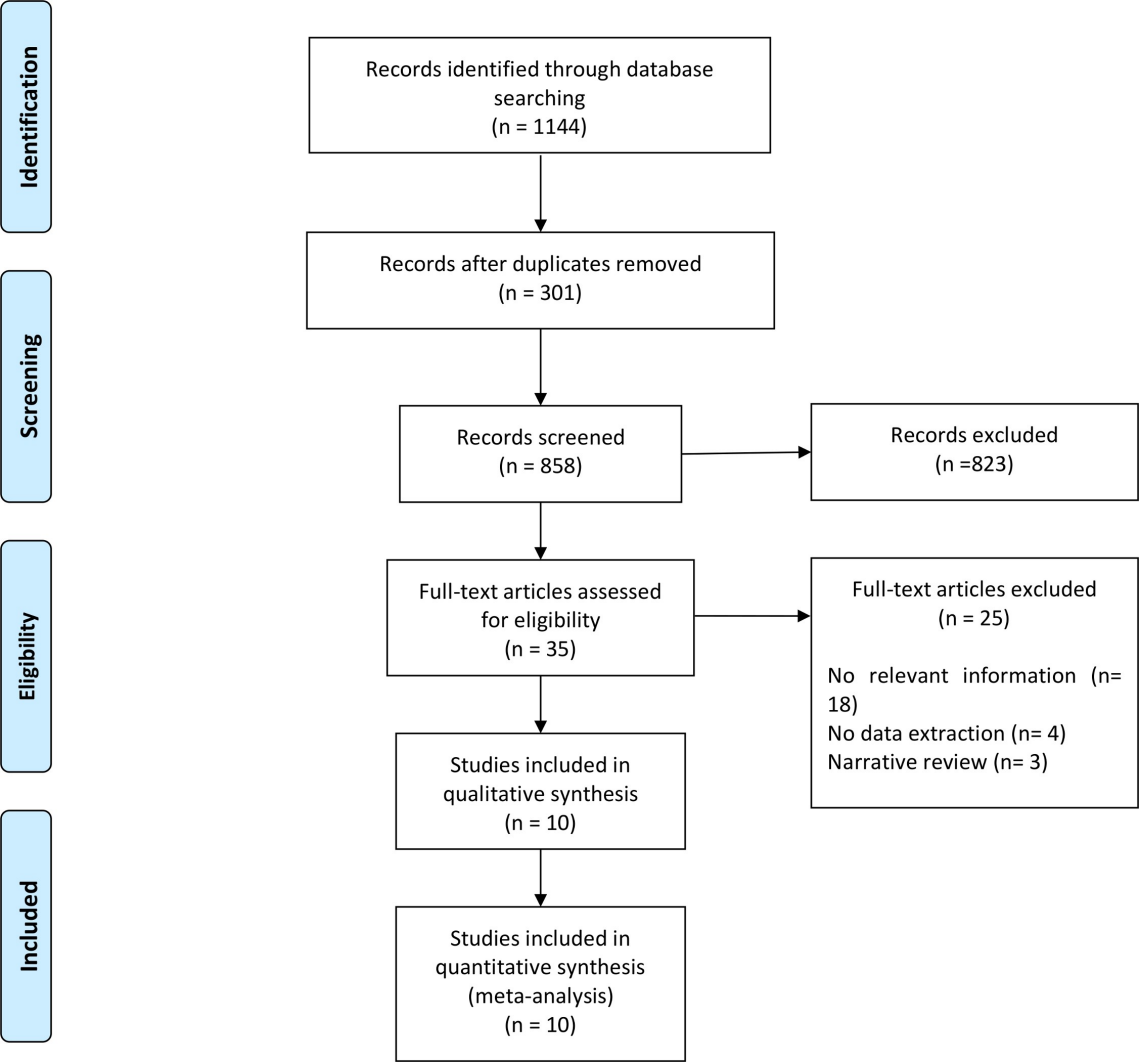
Flowchart of studies for inclusion in the meta-analysis.

The literature search identified 1,144 records. After screening titles and abstracts, 35 full-text articles were assessed for eligibility and 10 were included (Fig 1). Table 1 summarizes the main characteristics of the 10 studies. Five studies used a case-control design [13,23–26] and five were cohorts [14,16,27–29]. No clinical trials were found. Nine studies focused on very-low birth weight babies [13,14,16,23,25–29] and one considered the whole preterm population (gestational age <37 weeks) [24]. Most studies reported only infection as an outcome [13,14,25,27–29], two reported only NEC [23,24] and four included the three main outcomes of infection, NEC and mortality [14,16,26,29].

**Table 1:** Main characteristics of the studies analysed.

Study	Country	Study design	Population	Subject characteristics	Risk factors used	Statistics strategy	Outcome
Rojas et al. (2005) [27]	Colombia	Cohort prospective	Very Low Birth Weight	Birth weight: <2000g. Gestational age: <35 weeks	Maternal factors: age, fever (>38°C), prenatal antibiotics, prenatal steroids, premature ruptured membranes, type of delivery (vaginal, elective caesarean section, emergency). Infant factors: birth weight, gestational age, gender, mechanical ventilation, oxygen, postnatal antibiotics, catheters (central and non-central), parenteral nutrition and gastric tube for enteral nutrition.	Univariate and Multivariate logistic regression	Infection
Guillet et al. (2006) [23]	USA	Case-control	Very Low Birth Weight	Birth weight: 401g-1500g. Gestational age: not available	Maternal factors: not available. Infant factors: birth weight, gender, race, site of birth and Apgar score.	Univariate and Multivariate logistic regression	Necrotizing enterocolitis
Bianconi et al. (2007) [13]	USA	Case-control	Very Low Birth Weight	Birth weight: not available. Gestational age: not available	Maternal factors: not available. Infant factors: birth weight, gestational age, gender, length of stay in NICU, duration of total parenteral nutrition, pharmaceutical substances used and duration for central vascular catheters.	Univariate analysis	Infection
Afjeh et al. (2012) [28]	Iran	Cohort retrospective	Very Low Birth Weight	Birth weight: <1500g. Gestational age: <37 weeks	Maternal factors: age, fever (>38°C), prenatal antibiotics, prenatal steroids, premature ruptured membranes, type of delivery (vaginal, elective caesarean section, emergency). Infant factors: birth weight, gestational age, gender, Apgar score, intubation at delivery room, duration of mechanical ventilation, duration of oxygen therapy, postnatal antibiotics, chest tube, catheters (central and non-central), parenteral nutrition and gastric tube for enteral nutrition.	Univariate and Multivariate logistic regression	Infection
Terrin et al. (2012) [14]	Italian	Cohort prospective	Very Low Birth Weight	Birth weight: 401-1500g. Gestational age: 24–32 weeks	Maternal factors: age, fever (>38°C), prenatal antibiotics, prenatal steroids, premature ruptured membranes, type of delivery (vaginal, elective or emergency caesarean section). Infant factors: birth weight, gestational age, gender, Apgar, duration of mechanical ventilation, oxygen therapy, postnatal antibiotics, catheters (central and non-central), parenteral nutrition and gastric tube for enteral nutrition.	Univariate and Multivariate logistic regression	Infection, necrotizing enterocolitis and mortality
Bilali et al (2013) [24]	Greece	Case-control	Pre-term	Birth weight: not available. Gestational age: <37 weeks	Not available.	Univariate and Multivariate logistic regression	Necrotizing enterocolitis
Gupta et al. (2013) [25]	USA	Case-control	Very Low Birth Weight	Birth weight: <1500g Gestational age: <34 weeks	Maternal factors: age, fever (oral temperature >38°C), antibiotics, steroids, caffeine use, premature ruptured membranes, chorioamnionitis, type of delivery. Infant factors: birth weight, gestational age, gender and formula feeding.	Univariate analysis	Infection
Singh et al. (2016) [26]	Australia	Case-control	Very Low Birth Weight	Birth weight: < 1500g Gestational age: not available	Maternal factors: not available. Infant factors: birth weight, gestational age, gender, Apgar, patent ductus arteriosus, mechanical ventilation, oxygen therapy, continuous positive airway pressure, postnatal antibiotics, catheters (central and non-central), parenteral nutrition, type of feeding, gastric tube for enteral nutrition and length of hospital stay.	Univariate analysis	Infection, necrotizing enterocolitis and mortality
Romaine et al. (2016) [16]	USA	Cohort prospective	Very Low Birth Weight	Birth weight: <1500g. Gestational age (median): <32 weeks	Maternal factors: type of delivery (vaginal or caesarean section). Infant factors: birth weight, gestational age, gender, Apgar, mechanical ventilation, oxygen therapy, inotropic support and neutropenia.	Univariate analysis	Infection, necrotizing enterocolitis and mortality
Santana et al. (2017) [29]	Brazil	Cohort retrospective	Very Low Birth Weight	Birth weight (median): <1500g;.Gestational age (median): <34 weeks	Maternal factors: age, fever (oral temperature >38°C), prenatal care, premature rupture of membranes, type of delivery (vaginal or caesarean section), hypertension, Diabetes mellitus, gestational diabetes. Infant factors: birth weight, gestational age, gender, Apgar, duration of mechanical ventilation, oxygen therapy, duration of catheters (central and non-central), duration of parenteral nutrition and duration of gastric tube for enteral nutrition.	Univariate analysis	Infection, necrotizing enterocolitis and mortality

The risk of bias assessments are summarized in Tables 2 and 3, respectively. Overall, cohort studies had a lower risk of bias than case-control studies. The use of different criteria across studies for the selection of comparison groups (not exposed to H_2_RA) may have introduced a high risk of bias, especially among case-control studies.

**Table 2:** Assessment of study quality and risk of bias from case-control studies.

Study	Selection				Comparability	Exposure		
	Adequate case definition	Representativeness of cases	Selection of controls	Definition of controls	Case and control are comparable	Ascertainment of exposure	Same method of ascertainment for cases and controls	Non-response rate
Guillet et al., 2006 [23]	Yes	Yes	No	No	Yes	Yes	Yes	No
Bianconi et al., 2007 [13]	Yes	No	No	No	Yes	Yes	Yes	No
Bilali et al., 2013 [24]	Yes	Yes	No	Yes	Yes	Yes	Yes	No
Gupta et al., 2013 [25]	Yes	Yes	No	No	Yes	No	Yes	No
Singh et al., 2016 [26]	Yes	Yes	No	Yes	Yes	Yes	Yes	No

**Table 3:** Assessment of study quality and risk of bias from cohort studies.

Study	Selection				Comparability	Outcome		
	Representativeness of the exposure cohort	Selection of the non-exposed cohort	Ascertainment of exposure	Without outcome in the start	Cohorts are comparable	Assessment of outcome	Length of follow-up	Adequacy of follow-up
Rojas et al., 2005 [27]	No	No	Yes	No	Yes	Yes	No	Yes
Afjeh et al., 2012 [28]	No	No	Yes	No	Yes	Yes	Yes	Yes
Terrin et al., 2012 [14]	Yes	Yes	Yes	Yes	Yes	Yes	Yes	Yes
Romaine et al., 2016 [16]	Yes	Yes	Yes	Yes	Yes	Yes	Yes	Yes
Santana et al., 2017 [29]	Yes	Yes	Yes	Yes	Yes	Yes	Yes	Yes

**Fig 2:**
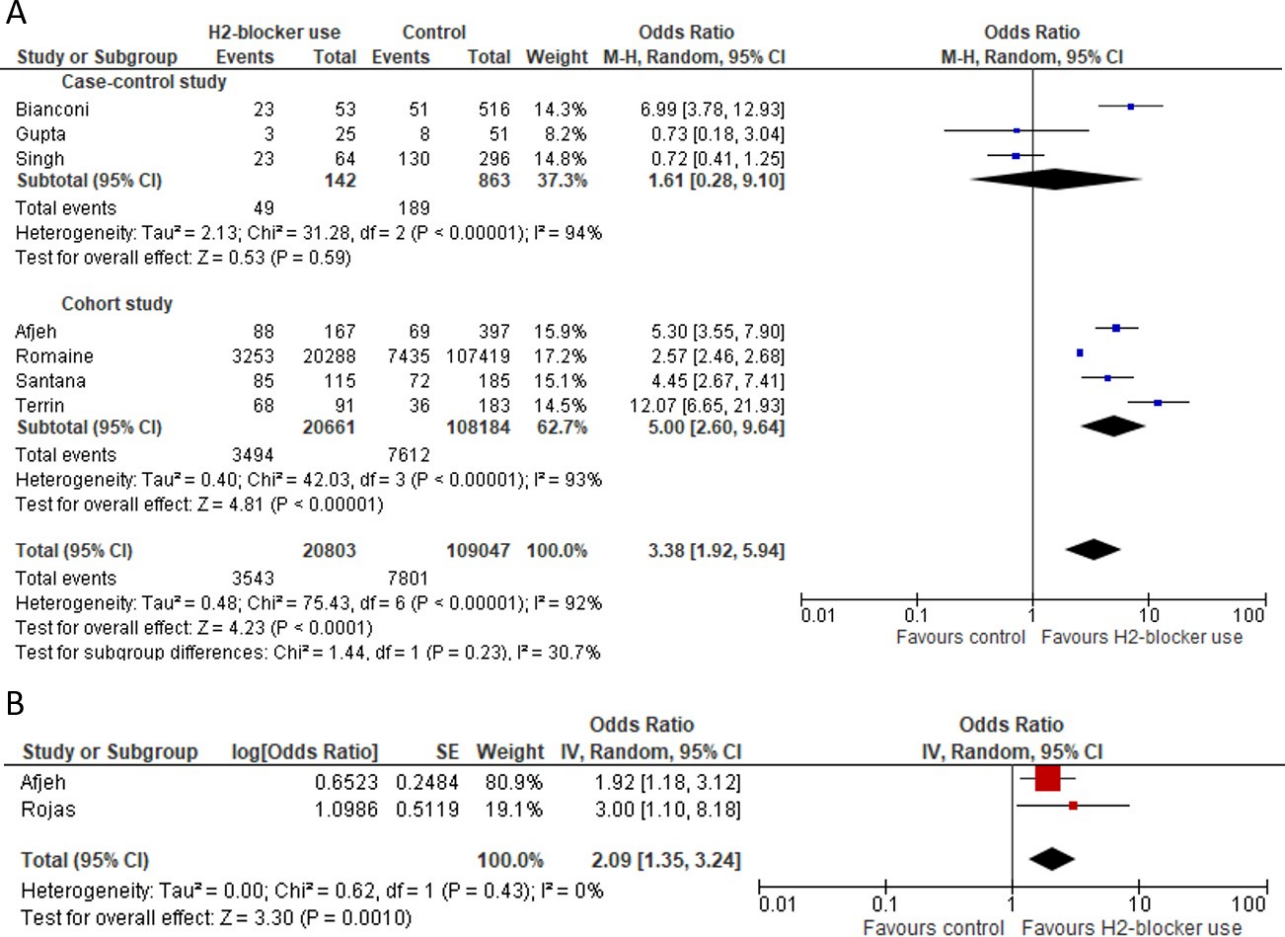
Forest Plot for infection outcome. A) unadjusted and B) adjusted pooled OR for infection.

Seven studies involving 129,850 subjects were included in the pooled OR estimation for infection. Of these, 3,543 (17.0%) of 20,803 neonates receiving H_2_RA had infections compared to 7,801 (7.2%) of 109,047 not exposed to H_2_RA, resulting in a pooled OR of 3.38 (95%CI: 1.92–5.94; P <0.001) (Fig 2A). There was substantial between-study heterogeneity (*I*^*2*^: 92%; 95%CI: 86.2% - 95.4%) and the sub-group meta-analysis demonstrated cohort studies influenced substantially the pooled OR. Based on studies [27,28] that had adjusted values, the pooled OR for infection was 2.09 (95%CI: 1.35–3.24; P<0.001) and the between-study heterogeneity was 0% (Fig 2B).

**Fig 3:**
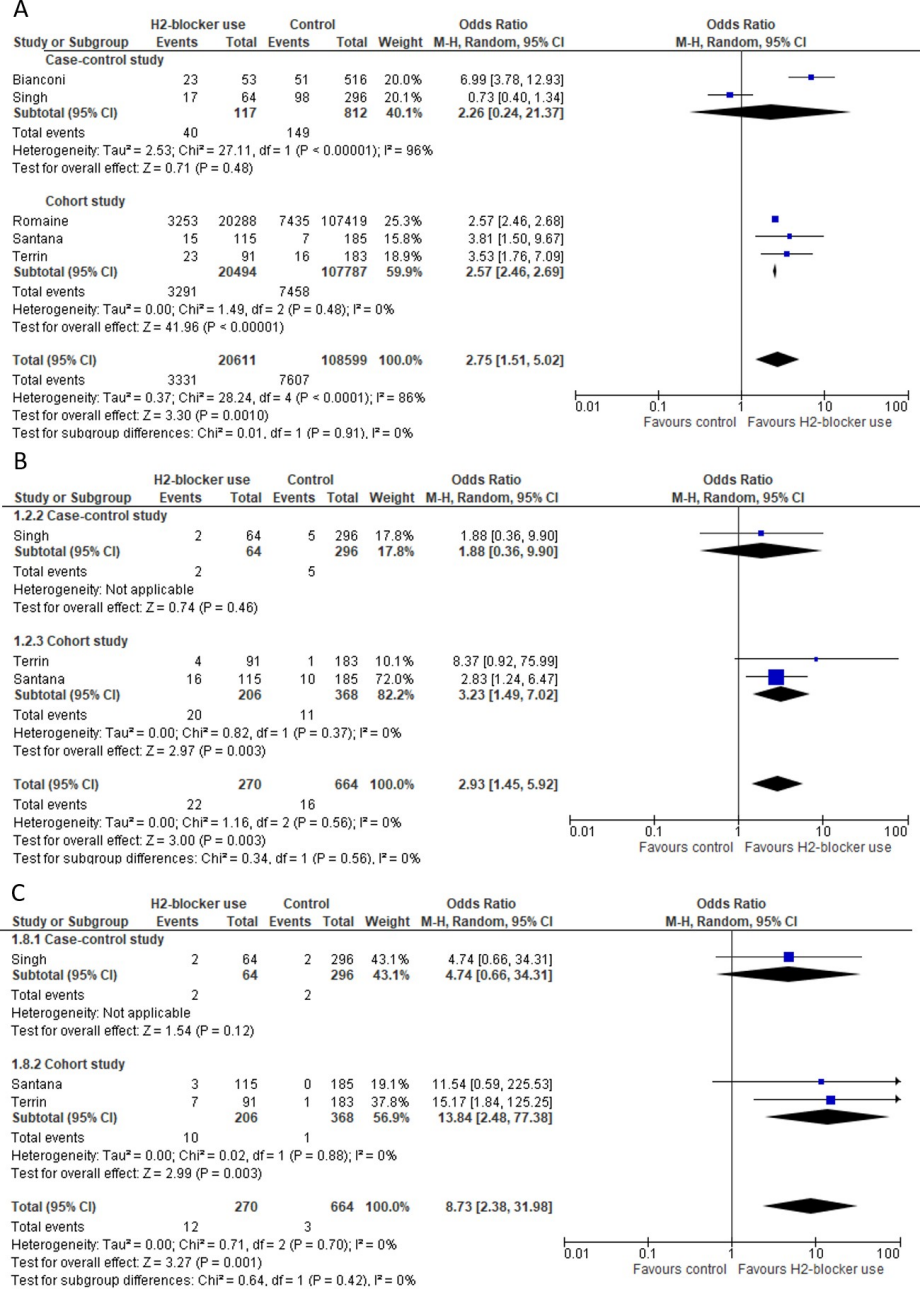
Forest Plot for sepsis, pneumonia and urinary tract infection. A) Unadjusted pooled OR for sepsis, B) Unadjusted pooled OR for pneumonia and C) Unadjusted pooled OR for urinary tract infection.

Some studies presented data for infection categories (Fig 3A–3C). Five studies had data for sepsis [13,14,16,26,29] and the pooled unadjusted OR was 2.75 (95%CI: 1.51–5.02; P: 0.001) (*I*^*2*^: 86%; 95%CI: 68.9%-93.6%). Subgroup analysis showed an association between sepsis and H_2_RA in cohort studies (OR: 2.57; 95%CI: 2.46–2.69; P <0.001) (*I*^*2*^: 0%; 95%CI: 0%-86.0%). Three studies reported pneumonia and urinary tract infections [14,26,29] with pooled ORs of 2.93 (95%CI: 1.45–5.92; P: 0.003) (*I*^*2*^: 0%; 95%CI: 0%-82.1%) and 8.73 (95%CI: 2.38–31.98; P: 0.001) (*I*^*2*^: 0%; 95%CI 0%-70.7%), respectively.

**Fig 4:**
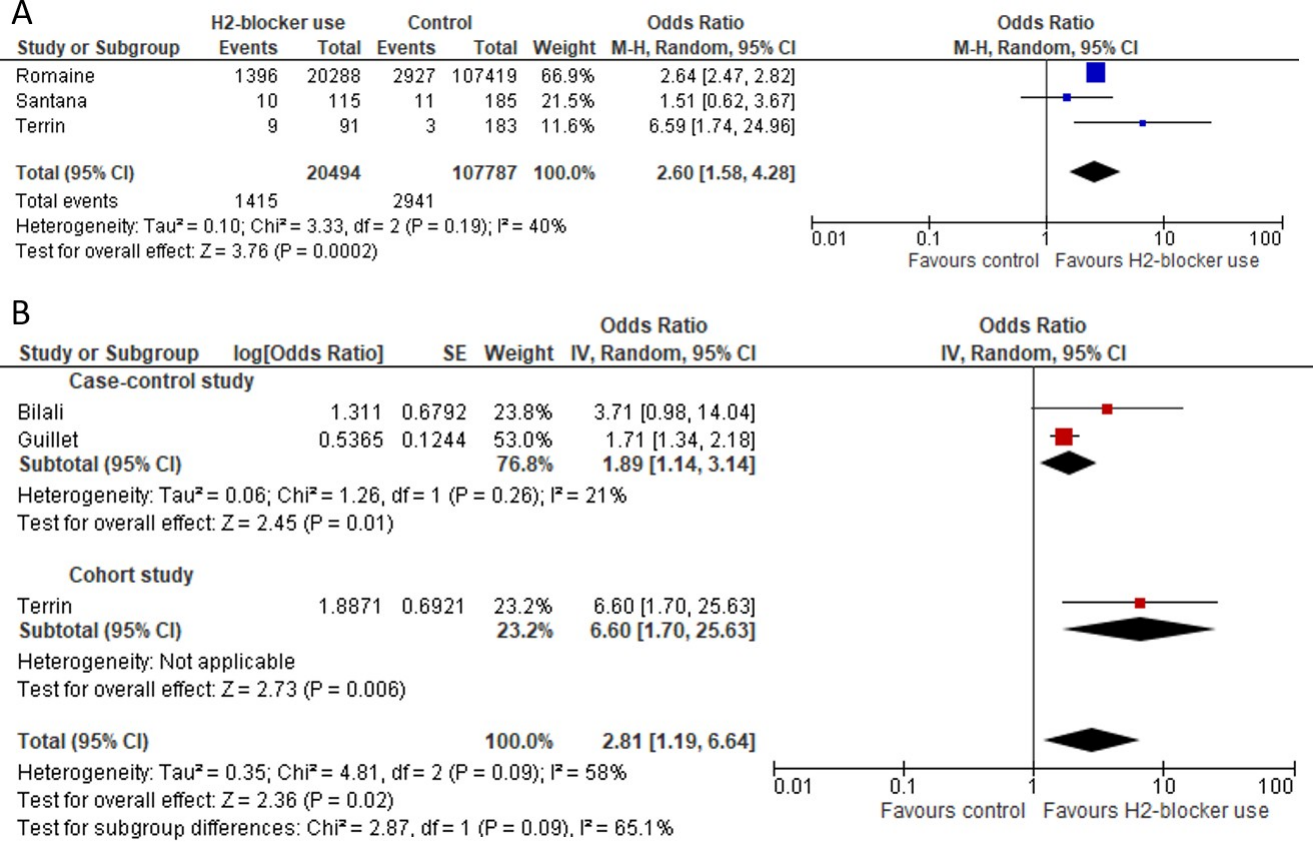
Forest Plot for necrotizing enterocolitis (NEC). A) Unadjusted and B) Adjusted pooled OR for NEC.

Unadjusted analyses from the three cohort studies evaluating NEC [14,16,29] indicated an association with H_2_RA (Fig 4A). Likewise, the meta-analysis of adjusted data [14,23,24] reported substantial association between NEC and H_2_RA (pooled OR: 2.81; 95%CI: 1.19–6.64; P: 0.02) (*I*^*2*^: 58%; 95% CI 0%-88.2%) (Fig 4B).

**Fig 5:**
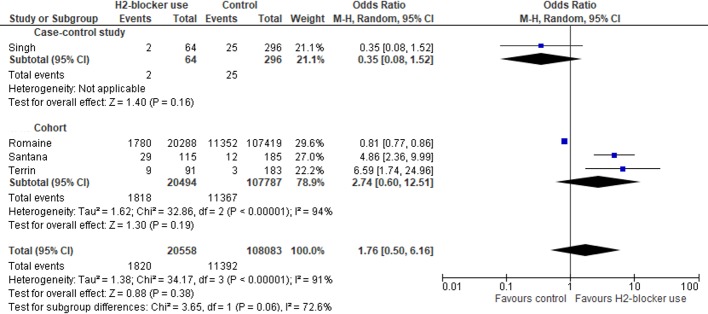
Forest Plot for mortality outcome.

Fig 5 shows that the use of H_2_RA was not associated with mortality (pooled OR: 1.76; 95%CI: 0.50–6.16; P: 0.38) (*I*^*2*^: 83%; 95% CI: 80.6%-96.0%).

## Discussion

The frequent *off-label* use of H_2_RA in neonates steered the European Medicines Agency and the US Food and Drug Administration to encourage studies on their safety and agencies responsible for drug regulations increased their search for information on their adverse effects in paediatric populations [2,14]. These initiatives has resulted in an increased number of studies, providing an opportunity to further ascertain adverse effects. In this systematic review and meta-analysis, we found that the use of H_2_RA in neonates is associated with increased risk of infections and NEC, but not with mortality.

Gastric fluid is an important non-immune barrier against pathogens [3] and the sustained inhibition of gastric acid secretion increases the pH and modifies the gastric microbiota [4,5]. The effects of H_2_RA administration are not restricted to the gastric pH, since they also increase the production of pro-inflammatory cytokines and reduce immunological responses to infection [8–10].

The main reasons for the prescription of H_2_RA in NICUs are the management of GERD and the prophylaxis and treatment of stress ulcers, usually caused by other drugs [11]. However, neonates receiving H_2_RA are more likely to exhibit GERD-like symptoms, resulting in a false perception that GERD is persisting, leading in turn to an increase of drug dosage and treatment duration [7]. Several studies have reported an average of 18 days between H_2_RA administration and the occurrence of infection [14,30] and 19 days for the occurrence of NEC [23], although one study from Brazil reported that infections started 6 days after H_2_RA use [29].

Only two studies evaluated whether the H_2_RA dosage was associated with unfavourable outcomes, as neonates who developed infection or NEC had received higher doses than children without these outcomes, but these differences were not statistically significant [14,29]. It is also noteworthy that the studies had used a wide H_2_RA dose ranges, making it difficult to identify a safe and effective dose.

This meta-analysis also found an association between H_2_RA use and pneumonia, sepsis, and UTI. It is well established that the inhibition of gastric acid secretion alters the bacterial ecology favoring gastric colonization by enteric bacteria and may facilitate microbial translocation across the stomach barrier [4,5], which may contribute to the development of pneumonia and sepsis [31]. Although the increased risk of pneumonia and sepsis involves gastric colonization with gram-negative bacteria [31], the results of the studies included in this review do not support this assertion. Although Rojas et al. (2005) [27] and Terrin et al. (2012) [14] studies reported a higher prevalence of gram-negative microorganisms, et al. (2007) [13] reported the same proportion of gram-negative and positive microorganisms among neonates receiving and not receiving H_2_RA.

The prolonged use of mechanical ventilation, central and peripheral catheters, parenteral nutrition and other devices in NICU is also associated with an increased risk of infection [32–37]. Although some studies in this meta-analysis controlled for these factors [14,23,24,27–29], = , the regression model still identified an association with H_2_RA.

Gestational age and low birth weight also increase the risk of nosocomial infections and NEC[38]. However, after controlling for these factors, the use of H_2_RA was independently associated with infection and NEC.

Although there is not enough information to assess whether H2RA increase the length of hospitalization, two studies reported a potential increase in hospitalization time [14,29], and further studies should be encouraged to generate this information in neonates.

The combined analysis of case-control and cohort studies did not show an association between H_2_RA use and mortality. However, there was large heterogeneity between studies and a paucity of quality data to examine the effect of H_2_RA and mortality, which may have resulted in a type II error. Further studies are needed to generate sufficient data to examine this association.

Our findings should be interpreted with caution as the number of studies showing the adverse effects for H2RA use in neonates admitted to NICU is small. All studies included were observational and treatments were not randomised. While the meta-analysis of cohort studies showed an association between H_2_RA and increased risk of infection, NEC and death, these associations were not fully evident in the case-control studies. Some studies had poor quality which may increase the risk of bias. Moreover, it was not possible to perform a funnel plot analysis due to the small number of studies. Finally, it was not possible to identify safe dosage thresholds or usage time for H_2_RA in neonates due to the scarcity of data and the wide variation reported between and within the studies.

Despite these limitations, current available evidence shows an association between the use of H_2_RA, the risk of infections and NEC in neonates. Further safety studies including well defined patient groups are needed to increase the evidence for their safe use in neonates and to support the development of guidelines by regulatory agencies. In the meantime, the use of H_2_RAin neonates must be stringently considered, when necessary.

## Supporting information

S1 Table Full search strategy. (DOCX)

S1 File MOOSE checklist for meta-analyses of observational studies. (DOC)
