# Supplementary material for: Association between histamine-2 receptor antagonists and adverse outcomes in neonates: A systematic review and meta-analysis
Source: PLoS One. 2019 Apr 4;14(4):e0214135. doi: 10.1371/journal.pone.0214135 (PMC6448909; doi:10.1371/journal.pone.0214135)
Supplement: S1 Table — (DOCX) [file pone.0214135.s001.docx]

**S1 Table. Full search strategy.**

| **#** | **Search strategy** |
| --- | --- |
| **1** | “Infant, Newborn” OR “Newborn Infant” OR “Newborn Infants” OR Newborns OR Newborn OR Neonate OR Neonates OR child OR children OR newborns OR "childhood disease" OR baby OR babies OR infant OR infants OR childhood OR toddler OR kids OR “young patient” OR boys OR girls OR young age OR pediatr* OR paediatr* OR "child death" OR "child care" OR "childhood mortality" OR "child hospitalization" OR "pediatric hospital" |
| **2** | “Histamine H2 Antagonists” OR “Receptor Blockaders, H2” OR “Blockaders, Histamine H2 Receptor” OR “Histamine H2 Receptor Blockaders” OR “Histamine H2 Blockers” OR “Blockers, Histamine H2” OR “H2 Blockers, Histamine” OR “Receptor Antagonists, Histamine H2” OR “Histamine H2 Receptor Antagonists” OR “Antagonists, Histamine H2” OR “H2 Antagonists, Histamine” OR “H2 Receptor Blockaders” OR “Blockaders, H2 Receptor” OR “Antihistaminics, H2” OR “H2 Antihistaminics” |
| **3** | death OR deaths OR died OR mortality OR mortalities OR fatal OR dying OR decreased OR "life threatening" OR "severe reaction" OR ICU OR "intensive care" OR emergency OR urgent OR fatalities OR enterocolitis OR “necrotizing enterocolitis” OR “neutropenic enterocolitis” OR “pseudomembranous enterocolitis” OR sepsis OR bacteremia OR fungemia OR “neonatal sepsis” OR “shock septic” |
| **4** | # 1 AND 2 |
| **5** | # 4 AND 3 |
